# Developing criteria for Cesarean Section using the RAND appropriateness method

**DOI:** 10.1186/1471-2393-10-52

**Published:** 2010-09-14

**Authors:** Rahim Ostovar, Arash Rashidian, Abolghasem Pourreza, Batool Hossein Rashidi, Sedigheh Hantooshzadeh, Hassan Eftekhar Ardebili, Mahmood Mahmoudi

**Affiliations:** 1 Department of Health Management and Economics, School of Public Health, Tehran University of Medical Sciences, Iran; 2 Department of Public Health, Yasouj University of Medical Sciences, Iran; 3 Knowledge Utilization Research Center, Tehran University of Medical Sciences, Iran; 4 Vali-e-Asr Reproductive Health Research Center, Faculty of Medicine, Tehran University of Medical Sciences, Iran; 5 Department of Health Education and Health Promotion, School of Public Health, Tehran University of Medical Sciences, Iran; 6 Department of Epidemiology and Biostatistics, School of Public Health, Tehran University of Medical Sciences, Tehran, Iran; 7 National Institute of Health Research, Italia Ave, Tehran, Iran

## Abstract

**Background:**

Cesarean section rates are increasing worldwide, and a rapid increase has been observed in Iran. Disagreement exists between clinicians about when to use cesarean section. We aimed to identify the appropriateness criteria for the use of cesarean section in Iran.

**Method:**

A consensus development study using a modified version of the RAND Appropriateness Method (RAM). We generated scenarios from valid clinical guidelines and expert opinions. A panel of experts participated in consensus development: first round via mail (12 members), second round face-to-face (9 members). We followed the RAM recommendations for the development of the scenario lists, rating scales, and statistical analyses.

**Results:**

294 scenarios relevant to cesarean section were identified. 191 scenarios were considered appropriate, of which 125 scenarios were agreed upon. The panel found cesarean inappropriate for 21% of scenarios, and 'equivocal' for 14% of scenarios.

**Conclusion:**

RAM is useful for identifying stakeholder views in settings with limited resources. The participants' views on appropriateness of certain indications differed with available evidence. A large number of scenarios without agreement may partly explain why it has been difficult to curb the growth in cesarean section rate.

## Background

Healthcare systems face significant challenges in response to changes in population needs and increasing costs. Studies show that a large proportion of healthcare offered may be inappropriate or unnecessary, ranging from 15 to 40 per cent in different countries and healthcare settings [1,2].

Different methods and tools have been developed to measure the appropriateness of care and develop valid criteria and recommendations for healthcare providers [3-5]. Most evidence originates from high income countries and it may not be possible for low and middle income countries to generate the expertise and resources required for the development of valid clinical guidelines [6]. Human interpretation plays an important role in the development of guidelines, and cultural and health system characteristics influence the way the evidence is interpreted and put into recommendation [4,7].

One of the most commonly performed surgical interventions is cesarean section. According to the WHO, a maximum of 15 per cent of deliveries have medical indications for cesarean section [8] and rates above this are unsuitable and unnecessary, imposing financial burden and clinical risks on patients and healthcare systems. The cesarean section rate has risen considerably over the past few decades: from less than 7% in the 1970 s to over 25% in 2003, causing major concerns for health policy makers [9]. Cesarean section comprised around 25, 26, 31, 31, 32 and 35 per cent of all deliveries in China, Canada, Australia, the United States, Taiwan and Italy respectively [10-13]. Figures are higher in South America: cesarean section comprises more than 50% of all deliveries performed in private hospitals in Chile, Argentina, Brazil and Paraguay [14]. The increase is multi-factorial and medical, legal, cultural, and economic concerns may have played roles in it [11,15,16].

In Iran, data published in 2005 suggested that cesarean section constituted 47% of all deliveries (around one million) in the country, 52% of deliveries in Tehran and 64% of deliveries in the private sector [17]. This was a significant increase over the 35% section rates reported for 2000 [18].

Little has been previously done in Iran for developing evidence-based criteria or consensus statements for the management of cesarean section. The most notable example was a protocol published by the Ministry of Health and Medical Education in 2004, but it lacked details and information expected in clinical guidelines [19]. It did not change the trend and the cesarean section rate in the country continued to rise despite the protocol. Recently, a quality improvement intervention in a Tehran hospital resulted in reduction of cesarean sections [20], suggesting that systematic approaches may yield positive outcomes.

Considering the magnitude of the problem in low and middle income countries in general, and in Iran in particular, we aimed to identify the appropriateness criteria for the use of cesarean section from the viewpoint of professional stakeholders in the country. The results of this study may benefit policy makers and clinicians in Iran as well as in other low and middle income countries.

## Method

The RAND Appropriateness Method (RAM) is an explicit approach for the assessment of the appropriateness of care. The method involves developing sets of clinical scenarios or criteria. Decisions are then made about the level of care or service that is appropriate for those scenarios and criteria. The method was designed in the 1980 s by the RAND and the University of California in Los Angeles (UCLA) and has been used in many studies in North America and Europe [4,21]. It has been frequently used for the development of appropriateness criteria in surgical care and investigative procedures [4,22-24]. We conducted our study in 2008 in Tehran. We followed a modified version of the RAM as explained below.

### Generating scenarios

First we searched the literature to identify available clinical practice guidelines and evidence summaries. We identified fourteen documents with relevant scopes [25-38]. We screened these documents and selected three guidelines and evidence summaries that provided a relatively comprehensive coverage of issues relevant to cesarean section [34-36]. The three selected documents had been developed as part of an established guideline development program or by recognized institutions. Then one author (AR) conducted a short workshop on development and appraisal of clinical guidelines. Three panel members and three authors used the validated Farsi translation of the AGREE (Appraisal of Guidelines for Evaluation and Research) tool (Additional file 1: Table S1) [39,40] to appraise the guidelines with comprehensive coverage and selected two clinical guidelines as a result [34,35]. In the next step, two obstetricians & gynecologists (BHR, SH), one midwife (FH) and a health service researcher (RO) extracted potential scenarios about the cesarean section from the selected clinical guidelines. We aimed to develop as short a list of scenarios as possible which were 'comprehensive', 'mutually exclusive' and 'homogeneous' [21]. Each scenario comprised of a few words or a short sentence capturing the main clinical features that identified the patients. The scenarios were categorized within main potential indications for cesarean section for further analyses and tabulation purposes.

### Panel members and setting

The panel members comprised of twelve individuals: nine obstetricians & gynecologists and three midwives. The panel members were from the Ministry of Health and Medical Education (one), four different public medical universities (nine), a non-profit medical school (one), and a private hospital (one). One participant exclusively worked in the private sector, and one exclusively in the public sector. The rest worked both in public and private hospitals.

Out of twelve invited members, ten responded to the first round. Those ten were then invited to participate in the second round, out of which nine participated.

### Consensus development

In the first round we sent the list of scenarios along with a summary of the clinical guidelines, the scoring system and the definitions to the panel members. The panel members were asked to give their opinions about each scenario for cesarean section on a scale ranging from one (totally inappropriate) to nine (totally appropriate). They were asked to consult the scientific resources provided for them while giving their opinions. The purpose of the literature review and the provision of evidence-based clinical guidelines and evidence summaries to the panel members was to provide them with an up-to-date summary of the best available evidence about the indications for cesarean section. The panels were not limited to the guidelines as they were required to use their own professional judgments as well as the evidence presented to them.

We then collated the views of the panel members and summarized the views in a format suitable for feedback so that each member received a summary of the panel view as well as a reminder of the scores that the member had assigned to each scenario. Then the panel members were invited to a second round, (face-to-face panel meeting), to view the feedbacks, and review and discuss their opinions. Nine members attended the second round (one day meeting from 8.00 to 17.00) and all the scenarios were reviewed and discussed. The panel members decided to add a limited number of further scenarios at this stage. Final decisions were recorded in specific forms.

As recommended by the RAM, we asked the panel members to rate the appropriateness of each indication (score them) based on their own professional judgment instead of what they perceived to be the views of other respected clinicians while considering the possible outcomes resulting from their decisions. The panel members were asked to consider average pregnant women presented to average clinicians in usual settings of care relevant to each indication discussed [21].

### Statistical analysis

We used the median scores for reporting the results of the first panel to the second panel. The scores were divided into three groups: appropriate (score = 7-9), equivocal (score = 4-6), and inappropriate (score = 1-3). If the median score fell into any of the above groups, it was considered as such (e.g. if a scenario's median score was 8, the scenario was considered as 'appropriate'). A further condition had to be met in order to reach agreement: if the minimum and maximum scores were ignored, all other scores must fall in the same scoring group. Additionally, we compared the rate of agreement between the two panels using weighed Kappa values and frequency charts.

## Results

We generated 276 scenarios for cesarean section in the scenario generation phase of the study. We divided the scenarios into thirteen indications as used in the selected guidelines [34,35] to make the assessment task easier for the panel members (Table 1) [21].

**Table 1:** Agreement on appropriateness of the scenarios for cesarean section according to the 13 main indications: first round of consensus development.

Cesarean Indications	Total number of scenarios	Appropriateness of scenarios			Agreement on appropriateness
			
		Inappropriate N (%)	Equivocal N (%)	Appropriate N (%)	
Repeat cesarean	18	0 (0)	3 (16.7)	15 (83.3)	11 (61)

Preterm delivery	49	10(20.4)	18 (36.7)	21(42.9)	4 (8.2)

Acute fetal distress	15	0 (0)	9 (60)	6 (40)	5 (33.3)

Chronic distress	21	1(4.8)	3 (14.3)	17 (81)	7(33.3)

Macrosomia	13	2 (15.4)	1(7.7)	10 (76.9)	3 (23)

Multiple gestation	28	0 (0)	10 (35.7)	18( 64.3)	5 (17.8)

Abnormal presentation (breech)	28	0 (0)	1(3.6)	27 (96.4)	17 (60.7)

Dystocia of soft tissue	14	0 (0)	8 (57.1)	6 (42.9)	1(7.1)

Cephalic Pelvic Disproportion (CPD)	24	0 (0)	8 (33.3)	16 (66.7)	14 (58.3)

Fetal anomaly	5	0 (0)	1(20)	4 (80)	0 (0)

Mother characteristics	41	16 (39)	12 (29.3)	13 (31.7)	10 (43.4)

Uterine anomaly	4	0(0)	2 (50)	2 (50)	2 (50)

Hemorrhage and premature rupture of membrane	16	2 (12.5)	1(6.2)	13 (81.2)	9 (56.2)

Total	276	31(11.2)	77 (27.9)	168 (60.9)	88 (31.9)

Table 1 shows that preterm delivery and mother characteristics comprised the highest proportion of potential scenarios of cesarean section. In total over 60 per cent of the scenarios were considered as appropriate, but the agreement was reached on 88 (31.9%) of them. Only 31 scenarios were inappropriate at this stage. Comparing the indications, 'abnormal presentation' and 'cephalic pelvic disproportion' contained the highest number of appropriate scenarios with agreement.

The panel members added a further 18 scenarios to the list in the 2^nd ^consensus development round, all of which were relevant to the 'chronic distress' indication. Table 2 shows the rate of appropriateness and agreement of panel members in the second round. It is seen that among the 294 scenarios of cesarean section, 191 (65%) were considered as appropriate, amongst which 125 (42.5%) scenarios were agreed upon. Table 3 provides examples of scenarios under each indication where the clinicians have agreed on the appropriateness of cesarean section.

**Table 2:** Agreement on appropriateness of scenarios for cesarean section according to the 13 main indications: second round of consensus development.

Cesarean indications	Total number of scenarios	Appropriateness of scenarios			Agreement on Appropriateness
		**Inappropriate** **N (%)**	**Equivocal** **N (%)**	**Appropriate** **N (%)**	

Repeat cesarean	18	1 (5.6)	1 (5.6)	16 (88.9)	10 (55.5)

Preterm delivery	49	18 (36.7)	10 (20.4)	21 (42.9)	12 (24.5)

Acute fetal distress	15	3(20)	1(6.7)	11(73.3)	8 (53.3)

Chronic distress	39	4 (10.3)	5 (12.8)	30 (76.9)	20 (51.3)

Macrosomia	13	1(7.7)	0 (0)	12 (92.3)	6 (46.2)

Multiple gestation	28	7 (25)	7 (25)	14 (50)	7 (25)

Abnormal presentation (breech)	28	0 (0)	0 (0)	28 (100)	22 (75.6)

Dystocia of soft tissue	14	5 (35.7)	4 (28.6)	5 (35.7)	1(7.1)

Cephalic Pelvic Disproportion (CPD)	24	1(4.2)	6 (25)	17 (70.8)	14 (58.3)

Fetal anomaly	5	1(20)	0 (0)	4 (80)	0 (0)

Mother characteristics	41	19 (46.3)	5 (12.2)	17 (41.5)	12 (29.3)

Uterine anomaly	4	0 (0)	0 (0)	4 (100)	2 (50)

Hemorrhage and premature rupture of membrane	16	3 (18.8)	1(6.2)	12 (75)	11(68.7)

Total	294	63 (21.4)	40 (13.6)	191(65)	125 (42.5)

**Table 3:** Examples of scenarios under each indication that the panel members agreed on the appropriateness *(inappropriateness) *of cesarean section.*

**Repeat cesarean ***with*	
Three or more previous cesarean (A) Classic scar (A)	Unknown scar (A) Previous rupture of uterine (A)
	
**Preterm delivery ***with*	
Down syndrome (*I*) Anencephaly (*I*)	Eclampsy <30 weeks of pregnancy (A) History of fever in previous cesarean due to infections (A)
	
**Fetal distress**	
Sever Decolman (A) Bradycardia (A)	Tachycardia (A) Decrease of variability (A)
	
**Chronic distress**	
IUGR^, BPS^# ^normal, pregnancy <30 weeks and Bishop score <5 (A) IUGR, BPS normal, pregnancy <30 weeks and Bishop score >5 (A) ADF^$^, IUGR, BPS normal, pregnancy 34-37 weeks (A) ADF, IUGR, BPS normal, pregnancy 30-34 weeks (A)	
	
**Macrosomia ***and*	
Diabetic mother, fetal weight > 4000 g (A) Parity > 5 (A)	Previous traumatic delivery (A) Post term > 41 weeks, Bishop appropriate (*I*)
	
**Multiple pregnancy**	
Twin, mono amnion with sonography (A) Twin, di-amnion, both non cephalic, normal weight and age (A) Twin, cephalic-non cephalic, both with normal weight (A) Twin, non cephalic-cephalic, both with normal weight (A)	
	
**Breech ***and*	
Decolman (A) Placenta previa (A)	Nuliparous mother (A) Unknown footling (A)
	
**Dystocia of soft tissues **	
Arrest of dilatation: delayed long phase (more than 3 hours in nullipara and more than 1 hour in multipara) with appropriate contraction, no CPD (A) Arrest of dilatation (more than 2 hours in nullipara & multipara) with appropriate contraction, no CPD (*I*) Failure to progress: nulliparous, insufficient dilatation of cervix ( < 1.2 cm/hr), appropriate contraction, no CPD (*I*) Failure to progress: multiparous, insufficient dilatation of cervix ( < 1.5 cm/hr), appropriate contraction, no CPD (A)	
	
**Pelvic dysfunctions and abnormal presentation of fetus**	
Inlet: posterior-anterior diameter <10 cm (A) Inlet: transversal diameter <12 cm (A) Inlet and mid pelvic contraction (A)	
	
**Fetal anomaly**	
Hydrocephalus fetus (A) Macrocephalus fetus (A)	NTD Open (A) Hydrops fetalis (A)
	
**Mother characteristics**	
> 12 kg weight increase in pregnancy (*I*) > 18 kg weight increase in pregnancy (*I*)	Sever coronary artery disease (A) Mother with asthma (A)
	
**Uterine anomaly**	
Previous surgery on uterine including opening the endometrial tissue (A) Pelvic contraction caused by rickets or trauma (A) Over distention of the uterine and need to end the pregnancy (A)	
	
**Hemorrhage and premature rupture of membrane**	
Rupture of membrane for <18 hours (*I*) Rupture of membrane for >18 hours and lack of labor (*I*) Rupture of membrane >5 hours in patients with HIV (A) Previa, fetus ≤34 week, light vaginal hemorrhage (*I*)	

*A: appropriate; I: inappropriate

^Intrauterine Growth Retardation, ^$^Brain Parenchyma **Sonography**, ^#^Advanced Dynamic Flow

Table 4 comprises only those 276 scenarios that were considered in both rounds and shows the effects of the second panel on the decisions made (weighted kappa value = 0.53). Comparing the round 2 results with round 1, we observed a reduction in the proportion of 'equivocal' scenarios (from 28% to 15%) with a similar increase in the proportion of 'inappropriate' scenarios (from 11% to 23%). In the case of appropriate scenarios, however, these differences were small (from 168 to 174 scenarios-Table 4).

**Table 4:** Comparison of appropriate scenarios of cesarean section in round 1 and round 2 panels for 276 scenarios.

		Panel 2			Total
			
		inappropriate	Equivocal	appropriate	
Panel 1	Inappropriate	28	1	2	31
		10.1%	.4%	.7%	11.2%
	
	equivocal	26	28	23	77
		9.4%	10.1%	8.3%	27.9%
	
	appropriate	8	11	149	168
		2.9%	4.0%	54.0%	60.9%

	Total	62	40	174	276
		22.5%	14.5%	63.0%	100.0%

Weighted kappa value = 0.53

## Discussion

In evidence-based medicine the question arises about what should be done if there is insufficient evidence for a procedure routinely performed in practice [41]. Formal consensus development (including RAM) provides a timely and efficient solution when evidence is insufficient [42] while questions remain about the validity of recommendations based on such methods [43]. Evidence-based clinical guidelines often lack flexibility and may not provide enough details for clinicians when making decisions about individual patients [44]. In our study, using RAM, we tried to overcome this limitation by developing scenarios representative of the patients seen by clinicians in practice.

To the best of our knowledge this is the first study that has used the RAND Appropriateness Method for cesarean section indications. In our study, cesarean section was considered to be appropriate in 191 (65%) potential scenarios, of which agreement was reached for 125 (42.5%) scenarios. As expected, the participants agreed on the appropriateness of using cesarean section in a large proportion of scenarios presenting as chronic distress, abnormal presentation, hemorrhage and premature rupture of membrane, and fetal anomaly. They also agreed on the appropriateness of over half of repeat cesarean scenarios.

After two rounds of consensus development there were still 106 scenarios in which no agreement was reached or the results remained equivocal. This may demonstrate the ambiguous nature of decision making on whether the cesarean section is indicated for an individual pregnant woman. It may partly explain why it has been so difficult to curb or slow the growth in cesarean section rate around the world [8,11-15,17,18].

### Advantages and limitations

Tan et al [43] have described the complexities and limitations of using RAM. Many of their criticisms equally apply to other consensus development methods, such as Delphi [45]. It should be noted that consensus methods are most useful where there are disagreements or variation in practice, and reliable evidence is limited. In these circumstances formal consensus methods are valuable and their use is inevitable.

There were heated discussions in our panel on whether it is indicated to conduct cesarean sections for repeat cesarean patients. Based on their personal experiences, members believed that the evidence on the benefits of trial of labor over repeat cesarean is dependent on a context with good quality pre-hospital and hospital care and may not apply to Iran's conditions. This was in contrast with the results of a systematic review of relevant evidence [46]. It concluded that repeat cesarean was not indicated as a routine practice, mostly based on non-randomized trials originating from high resource countries [46]. In our opinion, while high quality evidence on the issue is lacking, the panel members may have been somehow justified to doubt the benefits of trial of labor over previous cesarean section. A qualitative study in Canada reported that clinicians had similar concerns about trial of labour over previous caesarean section [16]. Further research originating from low and middle income countries is required.

We spent time on familiarizing the panel members with the method and attracting their valued cooperation. Membership of the panel involved open discussions of personal views and practices, and that certain practices might not be supported by evidence or by other panel members. We also selected the members from different backgrounds and settings to improve comprehensiveness of the views [47,48]. As an advantage, we used the AGREE tool for selection of the evidence sources [39]. The AGREE tool has been used extensively for appraising clinical practice guidelines for different conditions including obstetrics care [49]. Using the AGREE tool provided a chance for the panel to reach a shared understanding of the evidence before embarking towards consensus building.

RAM usually results in a long list of scenarios [48]. To ease the use of its results it may be possible to develop user-friendly software, or to categorize the scenarios into indications and packages in the format of clinical guidelines. The RAM guidelines suggest that on average 150-200 scenarios can be rated in an hour once the panel members are used to the process [21]. In our experience a dedicated team of panel members reviewed and rated 294 scenarios in one long-day second-round meeting.

We used the weighted kappa values to compare the results of the two rounds of the study. It should be noted that the interpretation of kappa values here differs from other agreement studies. In a consensus development study, the investigators seek to improve agreement via changes in the views of the panel members. Hence high values of kappa are not sought. Still its measurement is useful as it quantifies how the views have changed during the study.

### Implications

Our results will help decision makers in identifying misconceptions on the benefits of cesarean section and focusing their efforts on changing the views of the clinicians. For example the participants agreed that cesarean section was appropriate for many repeat cesarean and cephalic-pelvic disproportion scenarios, despite recent evidence and the recommendations of some evidence-based guidelines against use of section in these scenarios [34,35,46].

While our study focused on developing criteria for a wider use in the country, our approach may also benefit obstetricians and midwives working in hospitals to develop corporate strategies. It will require methodological support and group work, while it will help in generating shared views and understanding. In a way, the process will be similar to 'participatory guideline development' that has been shown to be effective in changing professional practice [50].

Other variables such as payment method, medico-legal issues and patient preferences may affect provider practice and views on conducting cesarean section [16]. According to previous studies different factors cause high cesarean rates in Iran. They include factors that affect women preference (e.g. increasing women level of education, employment and age at marriage and decreasing intended number of deliveries) [18,51-53], provider behavior and clinical factors (e.g. repeat cesarean, dystocia, CPD and physician preference) [51-53] and health system factors (e.g. health insurance coverage, delivery at private hospital) [51].

In Iran, as in many low and middle income countries, health system regulatory mechanisms are insufficient, and the fee-for-service payment provides further income if cesarean section is performed. It is also generally perceived that women prefer cesarean section over vaginal birth. In such context it is hardly surprising to see the current high rates of cesarean section. Also there is a growing culture of suing doctors because of malpractice claims, and this may fuel cesarean section rates as a form of 'defensive' medical practice. Hence our panel members may have been more lenient towards section (e.g. advocating repeat cesarean) than it might be observed in other countries.

The results of this study can contribute to the development of national guidelines for use in the country. Obviously the impact of implementing such guidelines will depend on many factors, including using effective implementation strategies [44]. The guidelines more likely affect provider and health system factors. They may also help clinicians in effective communication with pregnant women when they request cesarean section.

For certain scenarios, however, agreements on appropriateness may not result in reducing variation in practice. For example it may be easy to agree with cephalic-pelvic disproportion scenarios, but these are difficult to measure and implement in practice.

## Conclusions

The RAM should be used more widely in low and middle income settings and in other areas of healthcare or other patient groups where controversies exist or the practice varies. It also has the added value of developing a level of ownership by the providers if they see that their peers and relevant stakeholders are adequately represented in the process. The results of this study can be used for developing national guidelines, conducting research to assess whether the criteria are followed in practice, and whether their application can curb the growing rate of cesarean section in all countries.

## Competing interests

The authors declare that they have no competing interests.

## Authors' contributions

RO, AR and AP selected the topic and designed the study. RO and AR analyzed the data, interpreted the findings and wrote the first draft of the manuscript. AR and RO conducted the AGREE workshop and two rounds of the panel. BR, SH and RO participated in selection of panel members, developing indications, and selection of scientific references. The members of the panel discussed and rated the indications in two rounds. MM and HEA provided statistical and methodological advice. All the authors commented on the first draft of the manuscript. AR and RO revised the manuscript. All authors read and approved the final manuscript.

## Funding

The study received funding from the Deputy of Research of Tehran University of Medical Sciences, contract number 86-04-27-6592.

## Ethics approval

The study was approved by the Ethics Committee of the Tehran University of Medical Sciences

## Pre-publication history

The pre-publication history for this paper can be accessed here:

http://www.biomedcentral.com/1471-2393/10/52/prepub

## Supplementary Material

Additional file 1 **Table S1: AGREE criteria for appraising the quality of clinical practice guidelines**^**32**^ Click here for file

## References

[B1] Phelps EC The methodologic foundations of studies of the appropriateness of medical care N Engl J Med 1993 1329 1241 45 10.1056/NEJM199310213291707 8413392

[B2] Borowitz M Sheldon T Controlling health care: from economic incentives to micro-clinical regulation Health Economics 1993 2 201 204 10.1002/hec.4730020302 8275164

[B3] Hicks NR Some observations on attempts to measure appropriateness of care BMJ 1994 309 730 33 7950529 10.1136/bmj.309.6956.730 PMC2540824

[B4] Nicollier FA Vader JP Froehlich F Gonvers JJ Burnand B Development of appropriateness criteria for colonoscopy Int J Qual Health Care 2003 15 15 22 10.1093/intqhc/15.1.15 12630797

[B5] Liu X Miles A Evaluating payment mechanisms: how can we measure unnecessary care? Health Policy Plan 1999 14 409 413 10.1093/heapol/14.4.409 10787657

[B6] Rashidian A Adapting valid clinical guidelines for use in primary care in low and middle income countries Prim Care Respir J 2008 17 136 137 10.3132/pcrj.2008.00055 18695845 PMC6619894

[B7] Bernstein SJ Lazaro P Fitch K Aguilar MD Kahan JP Effect of specialty and nationality on panel judgments of the appropriateness of coronary revascularization: a pilot study Medical Care 2001 39 513 520 10.1097/00005650-200105000-00011 11317099

[B8] Fernando A José M B Caesarean section: the paradox Lancet 2006 368 1472 73 10.1016/S0140-6736(06)69616-5 17071266

[B9] World Health Organization CDS INAS Bulletins 1995-2003. Cited from: Christilaw JE. Cesarean section by choice: constructing a reproductive rights framework for the debate Int J Gynaecol Obstet 2006 94 262 268 10.1016/j.ijgo.2006.04.006 16842789

[B10] Herng Cl Sudha X Maternal age and the likelihood of a maternal request for cesarean delivery: a 5-year population-based study Am J Obstet Gynecol 2005 192 848 855 10.1016/j.ajog.2004.09.133 15746681

[B11] Sufang G Padmadas SS Fengmin Z Brown JJ Stones RW Delivery settings and caesarean section rates in China Bulletin of the World Health Organization 2007 85 755 762 10.2471/BLT.06.035808 18038056 PMC2636484

[B12] Chalmers B Kaczorowski J Darling E Heaman M Fell DB O'Brien B Lee L Cesarean and vaginal birth in Canadian women: a comparison of experiences Birth 2010 37 44 49 10.1111/j.1523-536X.2009.00377.x 20402721

[B13] Monari F Di Mario S Facchinetti F Basevi V Obstetricians' and midwives' attitudes toward cesarean section Birth 2008 35 129 135 10.1111/j.1523-536X.2008.00226.x 18507584

[B14] Belizean J Althabe F Burros F Alexander S Rates and implication of cesarean sections in Latin America: ecological study BMJ 1999 319 1397 1402 10574855 10.1136/bmj.319.7222.1397 PMC28283

[B15] Penna L Arulkumaran S Cesarean section for non-medical reasons Int J Gynaecol Obstet 2003 82 399 409 10.1016/S0020-7292(03)00217-0 14499986

[B16] Chaillet N Dube E Dugas M Francoeur D Dube ZJ Gagnon S Identifying barriers and facilitators towards implementing guidelines to reduce caesarean section rates in Quebec Bull World Health Organization 2007 85 791 797 10.2471/BLT.06.039289 PMC2636490 18038061

[B17] Ministry of Health and Medical Education The Fertility Health Assessment Program Family Health Section, Tehran 2005

[B18] Ahmad-Nia S Delavar B Eini-Zinab H Kazemipour S Mehryar AH Naghavi M Caesarean section in the Islamic Republic of Iran: prevalence and some sociodemographic correlates Eastern Mediterranean Health J 2009 15 1389 1398 20218129

[B19] Deputy for Health Affairs Managed care, Book 1: protocols numbered 1-25 2004 Ministry of Health and Medical Education: Tehran [in Persian]

[B20] Aghlmand S Akbari F Lameei A Mohammad K Small R Arab M Developing evidence-based maternity care in Iran: a quality improvement study BMC Pregnancy and Child Birth 2008 8 20 10.1186/1471-2393-8-20 PMC2443790 18554384

[B21] Fitch K Bernstein SJ Aguilar MD Burnand B LaCalle JR The RAND/UCLA Appropriateness Method: Users Manual 2001 ISBN: 0-8330-2918-5

[B22] Porchet F Vader JP Larequi-Lauber T Costanza MC Burnand B Dubois RW The assessment of appropriate indications for laminectomy J Bone Joint Surg 1999 81-B 234 39 10.1302/0301-620X.81B2.8871 10204927

[B23] McDonnell J Stoevelaar HJ Bosch JL Kahan JP The appropriateness of treatment of benign prostatic hyperplasia: a comparison of Dutch and multinational criteria Health Policy 2001 57 45 56 10.1016/S0168-8510(01)00127-0 11348693

[B24] Quintana JM Arostegui I Azkarate J Goenaga JI Guisasola I Alfageme A Diego A Evaluation by explicit criteria of the use of total hip joint replacement Rheumatology 2000 39 1234 41 10.1093/rheumatology/39.11.1234 11085803

[B25] The Society of Obstetricians and Gynecologists of Canada clinical practice guidelines Guidelines for vaginal birth after previous caesarean birth No 147 J Obstet Gynaecol Can 2004 26 660 70 15248936

[B26] Royal College of Obstetricians and Gynecologists Birth after previous caesarean birth Green-top Guideline No 45 2004

[B27] Royal College of Obstetricians and Gynaecologists Induction of labour Evidence-based Clinical Guideline No 9 2001

[B28] American Academy of Family Physicians Cesarean delivery in family medicine (Position paper) 2003

[B29] Royal College of Obstetricians and Gynaecologists Shoulder Dystocia Guideline No 42 2005

[B30] SHR Department of Reproductive Health and Research Family and Community Health Managing complications in pregnancy and childbirth: a guide for midwives and Doctors Geneva: WHO 2003

[B31] The Society of Obstetricians and Gynecologists of Canada clinical practice guidelines Guidelines for vaginal birth after previous Caesarean birth. No 155 (replaces guideline No 147) J Obstet Gynaecol Can 2005 27 164 174 15943001 10.1016/s1701-2163(16)30188-8

[B32] The Society of Obstetricians and Gynecologists of Canada clinical practice guidelines Guidelines for operative vaginal birth, No 148 2004

[B33] New Zealand Guidelines Group Care of women with breech presentation or previous Caesarean birth 2004

[B34] National Collaborating Centre for Women's and Children's Health Caesarean section: NICE clinical guideline London: the Royal College of Obstetricians and Gynecologists 2004

[B35] Task Force on Caesarean Delivery Rates Evaluation of cesarean delivery Washington DC: the American College of Obstetricians and Gynecologists 2000

[B36] Royal College of Obstetricians and Gynaecologists The management of breech presentation Guideline No 20b 2006

[B37] The Society of Obstetricians and Gynecologists of Canada clinical practice guidelines Diagnosis and management of placenta previa, No 189 2007

[B38] National Institutes of Health NIH state-of-the-science conference statement on cesarean delivery on maternal request 2006 23 1 1 29 17308552

[B39] The AGREE Collaboration Development and validation of an international appraisal instrument for assessing the quality of clinical practice guidelines: The AGREE project Qual Safety Health Care 2003 12 18 23 10.1136/qhc.12.1.18 PMC1743672 12571340

[B40] Rashidian A Yousefi-Nooraie R Moradi-Lakeh M AGREE instrument: validated Farsi (Persian) translation. 2007. Translated from: The AGREE Collaboration The Appraisal of Guidelines for Research & Evaluation (AGREE) Instrument. London: The AGREE Research Trust 2001

[B41] Black N Evidence-based surgery: a passing fad? World J Surg 1999 23 789 793 10.1007/s002689900581 10415204

[B42] Murphy MK Black NA Lamping DL Mckee CM Sanderson CFB Askham J Consensus development methods and their use in clinical guidelines development Health Technol Assessment 1998 2 3 9561895

[B43] Tan C Treasure T Browne J Utley M Davies CWH Hemingway H Seeking consensus by formal methods: a health warning J R Soc Med 2007 9 1 5 10.1258/jrsm.100.1.10 PMC1761668 17197680

[B44] Rashidian A Eccles MP Russell I Falling on stony ground? A qualitative study of implementation of clinical guidelines' prescribing recommendations in primary care Health Policy 2008 85 148 161 10.1016/j.healthpol.2007.07.011 17767976

[B45] Yousefi Nooraie R Rashidian A Keating JL Schonstein E Teaching evidence-based practice: the teachers consider the content J Eval Clin Practice 2007 13 569 575 10.1111/j.1365-2753.2007.00885.x 17683298

[B46] Cristina AR Vincenzo DA Maternal morbidity following a trial of labor after cesarean section vs elective repeat cesarean delivery: a systematic review with metaanalysis Am J Obstet Gynecol 2008 199 224 231 10.1016/j.ajog.2008.04.025 18511018

[B47] Coulter I Adams A Shekelle P Impact of varying panel membership on ratings of appropriateness in consensus panels: a comparison of a multi and single disciplinary panel Health Serv Res 1995 30 577 591 7591782 PMC1070076

[B48] Kahan GP Park RE Leap LL Bernstien SJ Hilborne LH Parker L Variations by specialty in physician ratings of the appropriateness and necessity of indications for procedures Med Care 1996 34 512 523 10.1097/00005650-199606000-00002 8656718

[B49] Appleyard TL Mann CH Khan KS Guidelines for the management of pelvic pain associated with endometriosis: a systematic appraisal of their quality BJOG 2006 113 749 57 10.1111/j.1471-0528.2006.00937.x 16827756

[B50] North of England Study of Standards and Performance in General Practice. Medical audit in general practice. I Effects on doctors' clinical behavior for common childhood conditions BMJ 1992 304 1480 1484 10.1136/bmj.304.6840.1480 1611372 PMC1882231

[B51] Farin Tatari P Afshari P Haghighi MH Survey of the factors affecting cesarean section in Mashhad hospitals,Iran J Ilam University Medical Sciences 2003 42-43 25 30 [in Persian]

[B52] Taavoni S Haghani H Mirzendedel S Vaginal delivery and cesarean section: comparative study of personal characteristics Middle East J Nursing 2007 1 1

[B53] Moini A Riazi K Ebrahimi A Ostovan N Caesarean section rates in teaching hospitals of Tehran: 1999-2003 Eastern Mediterranean Health Journal 2007 13 457 460 17684866

