# Supplementary material for: Developing criteria for Cesarean Section using the RAND appropriateness method
Source: BMC Pregnancy Childbirth. 2010 Sep 14;10:52. doi: 10.1186/1471-2393-10-52 (PMC2949786; doi:10.1186/1471-2393-10-52)
Supplement: Additional file 1 — Table S1: AGREE criteria for appraising the quality of clinical practice guidelines32 [file 1471-2393-10-52-S1.DOC]

Table S1: AGREE criteria for appraising the quality of clinical practice guidelines^32^

| **Scope & purpose**  The overall objective(s) of the guideline is(are) specifically described  The clinical question(s) covered by the guideline is(are) specifically described  The patients to whom the guideline is meant to apply are specifically described  **Stakeholder involvement**  The guideline development group includes individuals from all the relevant professional groups  The patients’ views and preferences have been sought  The target users of the guideline are clearly defined  The guideline has been piloted among target users  **Rigour of development**  Systematic methods were used to search for evidence  The criteria for selecting the evidence are clearly described  The methods used for formulating the recommendations are clearly described  The health benefits, side effects and risks have been considered in formulating the recommendations  There is an explicit link between the recommendations and the supporting evidence  The guideline has been externally reviewed by an expert panel prior to publication  A procedure for updating the guideline is provided  **Clarity & presentation**  The recommendations are specific and unambiguous  The different options for management of the condition are clearly presented  Key recommendations are easily identifiable  The guideline is supported with tools for application  **Applicability**  The potential organisational barriers in applying the guideline have been discussed  The potential costs implications of applying the recommendations have been considered  The guideline presents key review criteria for monitoring and/or audit purposes  **Editorial independence**  The guideline is editorially independent from the funding body  Conflicts of interest of guideline development members have been recorded |
| --- |
